# A pathway for skin NTD diagnostic development

**DOI:** 10.1371/journal.pntd.0012661

**Published:** 2024-11-25

**Authors:** Michael Marks, Sundeep Chaitanya Vedithi, Wendy W. J. van de Sande, Bruno Levecke, Anthony W. Solomon, Kingsley Asiedu, Camilla L. Ducker, Patrick Lammie, Daniel Argaw Dagne, Isra Cruz, on behalf of the WHO Diagnostic Technical Advisory Group on Neglected Tropical Diseases

**Affiliations:** 1 Clinical Research Department, Faculty of Infectious and Tropical Diseases, London School of Hygiene & Tropical Medicine, London, United Kingdom; 2 Hospital for Tropical Diseases, University College London Hospital, London, United Kingdom; 3 Division of Infection & Immunity, University College London, London, United Kingdom; 4 American Leprosy Missions, Greenville, South Carolina, United States of America; 5 Department of Medicine, University of Cambridge, Cambridge, United Kingdom; 6 Department of Medical Microbiology & Infectious Diseases, Erasmus Medical Centre, Rotterdam, the Netherlands; 7 Department of Translational Physiology, Infectiology and Public Health, Ghent University, Gent, Belgium; 8 Global Neglected Tropical Diseases Programme, World Health Organization, Geneva, Switzerland; 9 NTD Support Center, Task Force for Global Health, Atlanta, Georgia, United State of America; 10 National School of Public Health, CIBERINFEC, Instituto de Salud Carlos III, Madrid, Spain; Institute of Continuing Medical Education of Ioannina, GREECE

## Background

Among the more than 20 diseases and disease groups featured in the 2021–2030 neglected tropical diseases (NTDs) road map published by the World Health Organization (WHO), 10 present primarily with cutaneous manifestations: Buruli ulcer, cutaneous leishmaniasis and post-kala-azar dermal leishmaniasis, mycetoma, chromoblastomycosis and other deep mycosis, leprosy, lymphatic filariasis, onchocerciasis, scabies and other ectoparasites, and yaws. These are sometimes referred to as skin NTDs. Skin NTDs hinder individuals’ quality of life through pain, deformity, loss of function, stigma, social discrimination, isolation, and loss of livelihoods. As a result, the 2021–2030 NTD road map identified skin NTDs as a priority area of work. To assist national programmes, in 2022, WHO released a companion document to guide integrated approaches to their detection, diagnosis, management, and control [1].

Skin NTDs pose a significant public health challenge globally, with the highest burden found in sub-Saharan Africa [2]. Despite a 1991 World Health Assembly resolution to eliminate leprosy as a public health problem by 2000, leprosy remains a concern, with current efforts focused on early detection, treatment, disability management, and stigma reduction. Buruli ulcer affects mainly children and requires early detection, antibiotics, wound care, and surgery. Yaws also affects mainly children, with mass drug administration and healthcare access the keys to its eradication. Cutaneous leishmaniasis necessitates early detection, (toxic) anti-parasitic drugs, topical therapies, and vector control. Lymphatic filariasis requires mass drug administration and morbidity management for hydrocele and lymphoedema. For interruption of transmission of onchocerciasis, WHO recommends mass drug administration of ivermectin and vector control in some areas. Scabies is prevalent worldwide, especially in overcrowded settings and requires early diagnosis and treatment. Mycetoma needs early diagnosis, differentiation between bacterial and fungal aetiology to guide medication, and often surgery. Tungiasis requires prevention through improved living conditions and health education, along with early detection, flea extraction, and wound care.

From 2019 to 2023, WHO’s Diagnostic Technical Advisory Group (DTAG) for NTDs established a Skin NTDs subgroup and oversaw the development of target product profiles (TPPs) for multiple skin NTDs [3,4].

WHO’s *Global Report on Neglected Tropical Diseases 2024* notes a progressive increase in the adoption and execution of integrated strategies targeting skin NTDs. This momentum is also reflected by the change in name of the WHO Laboratory Network for Buruli ulcer in Africa to become the WHO Skin NTDs Laboratory Network [5,6]. However, there has been limited progress in the development of essential diagnostics necessary to achieve agreed road map objectives for these diseases [5,6]. According to the latest G-FINDER report on investment in neglected diseases research and development (R&D) [7], 2022 saw mixed trends in funding for skin NTDs, with diagnostic funding remaining scarce. Leishmaniasis experienced its third consecutive year of decline, dropping by 18%, with minimal diagnostic funding ($0.5 million) awarded. In contrast, leprosy R&D funding increased for the second year in a row, rising by $3.7 million to $14 million, though less than 3% of this total was allocated for diagnostics. Scabies R&D funding remained stable at $1.9 million, with no specific allocation for diagnostics. Buruli ulcer R&D funding decreased by 7.7% to just under $0.6 million, with a significant cut in diagnostic funding, down 41% ($65,000). Mycetoma funding dropped by over a third to $0.5 million after being steady in previous years. Yaws received no funding for basic research or diagnostics, but $0.5 million was provided for diagnostic R&D through the EDCTP across different projects. Overall, global funding for these skin NTDs remained under 4% of the global investment in neglected diseases R&D (3,931.24 USD millions), which is low compared to the 34%, 18%, and 15% dedicated to HIV, TB, and malaria, respectively.

The present document outlines the next steps recommended by the Skin NTDs subgroup of the DTAG to operationalise individual disease TPPs and to advance the NTD road map approach of scaling up integrated approaches to skin NTDs.

## The diagnostic pathway for skin NTDs

**Fig 1:**
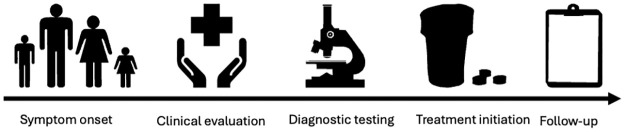
A typical journey for a skin NTD patient. A patient develops symptoms and ideally seeks healthcare in a timely fashion at the primary care level. If an appropriate clinical evaluation is made, a clinical diagnosis may be made (for most non-skin NTD diagnoses) or a decision taken that diagnostic testing is required. In the latter case, appropriate tests may be available at the primary care facility or onward referral may be required. Currently, patients experience bottlenecks at every stage of this journey.

Integrated clinical and laboratory diagnostic services are a cornerstone of the NTD road map. A typical patient journey entails multiple steps (Fig 1). Enhanced diagnostic strategies are required across this journey. Two critical junctures are (i) the initial clinical assessment of the patient; and (ii) confirmatory diagnostic testing. This process must be supported by strong reference laboratory capacity.

### Initial clinical evaluation

Skin changes associated with skin NTDs may facilitate early diagnosis based on history and examination alone. This requires accurate clinical diagnosis from a broad differential diagnosis which includes both the skin NTDs and other common skin conditions. Unfortunately, there are major barriers to achieving accurate and timely diagnosis of skin NTDs in most health systems in low- and middle-income countries. There is a substantial skill gap with an absence of highly trained dermatologists to guide care for most patients with skin NTDs, while mid- and lower-level healthcare workers (HWs) have limited training, inadequate access to diagnostics or specialist expertise, and the broad spectrum of skin conditions to consider.

There are a range of strategies that might improve the quality of initial clinical assessment, including training packages and supported approaches such as mobile phone apps, teledermatology and artificial intelligence (AI) tools. Several such tools are in use or development, including the WHO SkinNTD app. However none of these tools have yet been robustly validated either for their diagnostic performance or their impact on training. With the rising prevalence of ownership of mobile phones and other digital technologies across Africa, the Africa Centres for Disease Control and Prevention recognises a unique opportunity to capitalise on this potential and enhance health outcomes [5]. Smartphone applications offer a swift, secure, and user-friendly solution for efficiently collecting, integrating, storing, and safeguarding patient data from various sources. This facilitates the integration of digital technologies into healthcare systems and has the potential to revolutionise the delivery of health services, improving accessibility, quality of care, accountability, and cost-effectiveness.

### Confirmatory diagnostic testing

Most skin NTDs cannot be diagnosed by clinical examination alone and confirmatory laboratory diagnostic testing is needed. Implementing timely and effective diagnostics can facilitate early intervention. Many of the current diagnostics for skin NTDs demand sophisticated laboratory infrastructure, making them unsuitable for peripheral health system implementation [2,8]. WHO has developed TPPs for multiple skin NTDs. While these are critical in describing desired performance characteristics, it is important to recognise that in many settings the range of possible diagnoses for any given presentation extends beyond a single skin NTD, and beyond a simple differential diagnosis involving skin NTDs alone.

## Individual disease TPPs

Table 1 outlines the use cases that were the focus of the skin NTD TPPs. Five of these conditions share key common features including, notably, that the public health focus is on the identification and management of individual cases. For yaws, mycetoma, Buruli ulcer, and cutaneous leishmaniasis, the TPPs are focused on initial diagnosis and selection of appropriate therapy. For leprosy, there are 2 TPPs [4]. In considering leprosy within this document, we focus predominantly on the first use case (confirmation of leprosy in individuals with clinical signs) as it aligns most closely with the other skin NTD TPPs and is focused on supporting early patient diagnosis. As such these 5 TPPs are well aligned which should facilitate integrated approaches. Scabies has some important differences, including that it is considerably more common than any other skin NTD, clinical diagnosis has been demonstrated to have reasonable performance and the major strategy advocated for control is mass drug administration [9]. However, individual diagnosis and management of scabies remains an important component of an effective control programme. Notwithstanding these points, an ideal skin NTD test kit for both clinical diagnosis and confirmatory testing would include scabies.

**Table 1:** Target product profiles for skin neglected tropical diseases developed by the Diagnostic Technical Advisory Group of the World Health Organization.

Disease	Use-cases	Common characteristics
Buruli ulcer	• Diagnosis at PHC level	Sensitivity 90%–95% Specificity ≥95% Turnaround time 1 h–1 day Cost <USD 5 No cold chain required
Cutaneous leishmaniasis and PKDL	• Point of care test	
Leprosy	• Confirmation of leprosy in individual with clinical signs • Detection of asymptomatic infection	
Mycetoma	• Diagnosis at PHC level	
Scabies	• Diagnostic to start mass drug administration • Diagnostic to stop mass drug administration	
Yaws	• Identification of a case of yaws ● Detection of azithromycin resistance	

### Multiplex platforms

Most of the skin NTD TPPs do not directly address a requirement for multiplexing as this was not in scope during the development of disease-specific TPPs. The mycetoma TPP addresses the requirement to identify multiple causative agents, implying a need for multiplexing but not necessarily multiplexing with diagnostic tests for other NTDs. There would be clear advantages to developing integrated multiplex tests or platforms capable of delivering diagnostics for a range of skin NTDs, either in singleplex or multiplex, as required by local epidemiology. Multiplex platforms that would facilitate parallel testing of multiple different skin NTDs and detection of antimicrobial resistance markers would be particularly valuable.

**Fig 2:**
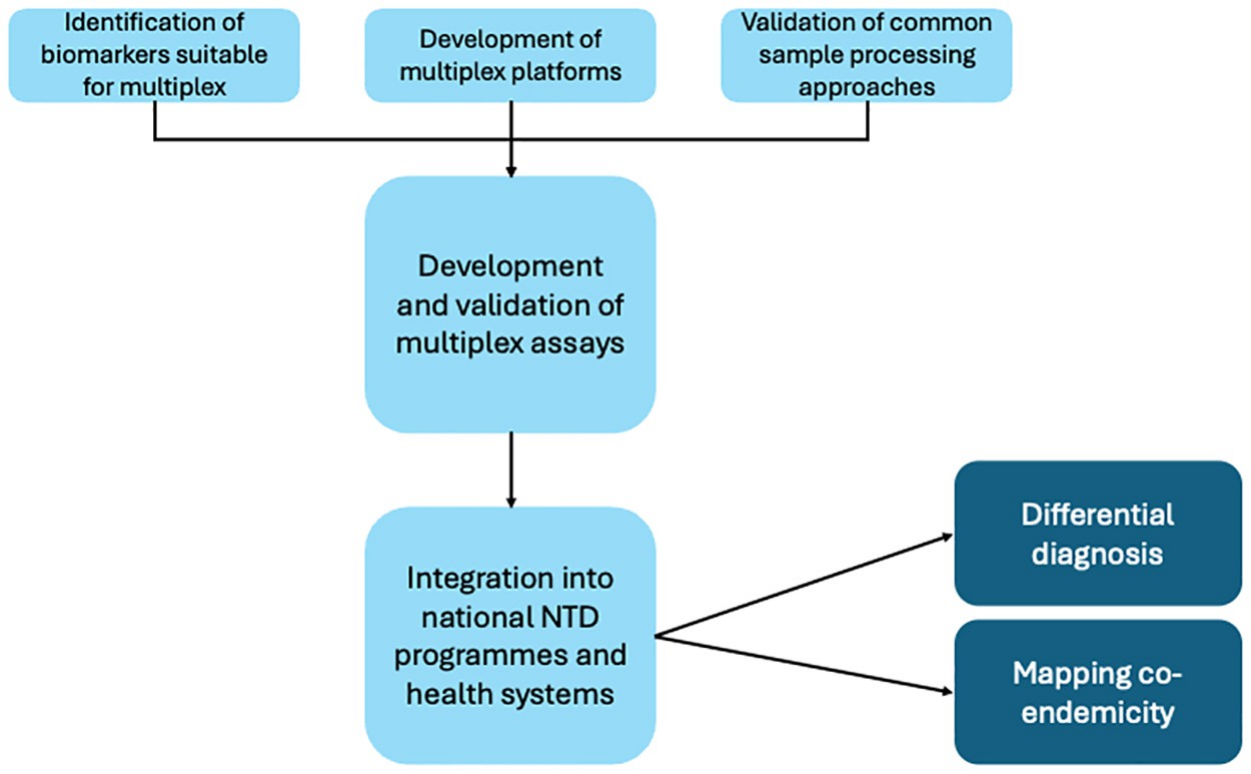
Exploration of multiplex platforms is recommended, as they would add significant value to the diagnostic landscape for skin NTDs. This aligns with the aims of the Skin NTD LABNET, which seeks to harmonize procedures for multiple skin NTDs as part of an integrated approach. While further development work is required, the advantages of multiplex platforms include the ability to perform differential diagnosis panels and disease mapping, offering a comprehensive and efficient diagnostic solution.

This requirement has received endorsement from WHO’s DTAG, which highlighted several advantages in employing multiplex platforms for diagnosing skin NTDs [10]. Development of these platforms may require research, development, and validation work, but will facilitate the creation of a comprehensive differential diagnosis panel and aid in the mapping of co-endemic diseases (Fig 2). Broadly, open platforms (i.e., platforms that allow new tests to be added) are preferred over closed platforms (i.e., platforms on which the tests that can be performed are restricted by the manufacturer).

Molecular tools capable of detecting pathogen nucleic acid in host tissues have shown high sensitivity and specificity for detecting the corresponding infections [11]. Recent advances in column-based DNA capture technologies have significantly improved the detection limits for identifying pathogen DNA in human skin tissue samples. For instance, preliminary data from Biomeme’s Franklin qPCR suggests the ability to capture DNA from as few as one bacterial cell [12]. The detection and quantification of pathogen DNA can aid in the confirmatory diagnosis of skin NTDs in individuals presenting ambiguous or nonspecific clinical signs. A nucleic acid amplification test (NAAT) system that requires minimal resources at point-of-care is highly desirable; such a system would increase the likelihood of accurate diagnosis of these diseases at the periphery of the healthcare system in resource limited settings.

Miniaturised and field-compatible NAATs provide a pathway to embed diagnostic excellence at laboratory, hospital, and field levels to assist in the confirmatory diagnosis of skin NTDs. Such systems would not only help prevent misdiagnosis everywhere but also support confirmation of cases in endemic areas where clinical expertise is scarce or declining; in the latter context, for diseases targeted for elimination or eradication, the consequences of overlooking a diagnosis may be particularly profound. For example, several portable, field-compatible NAAT machines and corresponding reagents have now become available [13]. Many systems offer a closed cartridge system for quick, contamination-free nucleic acid extraction. Both reagents and assays are long-lasting at ambient temperature. Many of these platforms can be linked to smartphones, facilitating offline testing followed by data upload onto a cloud-based portal, enabling remote monitoring, training, and collation, management and application of diagnostic data. This set of characteristics makes them potentially suitable for point-of-care or field-friendly diagnosis of various diseases affecting humans and animals.

## Tools employing artificial intelligence

To evaluate AI-based diagnostic technologies for skin NTDs diagnosis in diverse clinical and community settings, a comprehensive approach will be essential. This would involve conducting clinical validation studies in various geographical locations, considering local factors such as disease epidemiology and healthcare infrastructure. As part of these trials, active case-finding activities should be implemented within communities alongside enrolment at specialised health facilities. This approach might allow a broad range of both NTD and other skin conditions to be recruited in community settings while more advanced or complex skin-NTD cases recruited from specialised facilities. Prior to evaluation work, it is crucial to provide capacity building and standardised protocol training to the HWs involved.

Validation studies should compare different diagnostic approaches, including AI-based systems, existing digital tools like mobile applications, against the current reference standard of dermatologist-supported expert diagnosis. Discrepancies between diagnostic methods should be categorized based on their impact on treatment or management decisions.

Additionally, it will be important to solicit qualitative feedback from stakeholders such as program officers, healthcare workers, patients, and community members. This could be collected through focus group discussions and collection of quantitative data from system usability surveys, providing insights into the technology’s usability and effectiveness, and allowing progressive system improvement. Finally, there are important considerations about data ownership and sharing and ensuring adequate capacity for development of AI-based technologies within skin-NTD endemic countries.

## Reference laboratory networks

Strong reference laboratory networks are required to underpin decentralised diagnostics. In recent years, the WHO Buruli ulcer LabNet has supported reference laboratories in countries of West and Central Africa. Activities have included alignment and simplification of standard operating procedures, optimising reagent procurement and establishing an external quality assurance scheme to assure high-quality diagnostic performance in the region. This network has recently expanded and been re-named the WHO Skin-NTD LabNet, reflecting a broadening of its remit. The network is now focused on repeating similar steps for other skin NTD diagnostic tests. Expansion of this network or establishment of similar networks in other regions could play a critical role in supporting adoption of high-quality diagnostic testing for skin NTDs within national programmes.

## Next steps

Based on the considerations outlined above, the WHO DTAG Skin NTDs subgroup has identified the following actions as recommended next steps to advance diagnostic platforms for skin NTDs.

*Multiplex platforms:*

1. Identify multiplex diagnostic platforms suitable for skin NTDs programme use.
2. Undertake validation studies of diagnostic performance of multiplex platforms.
3. Perform real-world evaluations and integration of multiplex platforms into national NTD programmes.

*Artificial intelligence tools:*

1. Decision tool identification: identify candidate decision tools, including AI, teledermatology, and training programs, that are suitable for use in skin NTD programmes.
2. Data and collaboration: create robust data sets from diverse populations to train AI models and foster collaborations between technologists, healthcare providers, and policymakers to support the implementation and scaling of AI diagnostic tools. Ensure appropriate data-sharing for use of AI tools.
3. Algorithm development and integration: develop and refine machine learning algorithms for accurate identification and classification of skin NTDs from images.
4. Accessibility and training: ensure AI diagnostic technologies are affordable, accessible, and user-friendly for healthcare workers in endemic areas, providing necessary training and support.

*Skin NTD laboratory networks*

1. Develop and standardise standard operating procedures for all skin NTD diagnostic assays.
2. Develop and roll out external quality assurance systems for skin NTD diagnostic assays.
3. Expand or replicate the existing skin-NTD LabNet platform to cover all skin NTD-endemic countries.

The WHO DTAG skin NTDs subgroup has laid out a comprehensive plan to advance diagnostic platforms for skin NTDs, focusing on multiplex platforms, AI tools, and the expansion of laboratory networks. These recommended actions underscore the need for collaboration and innovation to enhance diagnostic capacities and improve health outcomes in NTDs endemic regions.

## Disclaimer

The authors alone are responsible for the views expressed in this article and they do not necessarily represent the views, decisions, or policies of the institutions with which they are affiliated.
